# Editorial: Language beyond Words: The Neuroscience of Accent

**DOI:** 10.3389/fnhum.2016.00639

**Published:** 2016-12-20

**Authors:** Ignacio Moreno-Torres, Peter Mariën, Guadalupe Dávila, Marcelo L. Berthier

**Affiliations:** ^1^Department of Spanish Language, University of MalagaMalaga, Spain; ^2^Department of Linguistics and Literary Studies, Clinical and Experimental Neurolinguistics, Vrije Universiteit BrusselBrussels, Belgium; ^3^Department of Neurology and Memory Clinic, ZNA Middelheim General Hospital AntwerpAntwerp, Belgium; ^4^Cognitive Neurology and Aphasia Unit and Cathedra ARPA of Aphasia, Centro de Investigaciones Médico-Sanitarias, Instituto de Investigación Biomédica de Málaga, University of MalagaMalaga, Spain; ^5^Department of Psychobiology and Methodology of Behavioural Sciences, Faculty of Psychology, University of MalagaMalaga, Spain

**Keywords:** accent, foreign accent syndrome, neuroscience, psychiatric disorders, neuroimaging

## Introduction

Speakers differ not only in the number of languages they master, but also in the accents they impart. Indeed, accent is an essential component of our identity to the extent that in many cases our social adscription to specific groups and our judgements about others are based on accent. The relevance of accent was often dismissed by many linguists for a large part of the twentieth century. However, today it is widely acknowledged that spoken language does not exist without an accent. There are, however, some circumstances under which speakers are unable to modulate accent properly. In fact, many late learners of a second language are unable to acquire the native-like accent in the new language, and some individuals with discrete brain lesions in the speech production network or neuropsychiatric problems may change or lose their regional accent or acquire a peculiar accent, which gives raise to what we know as foreign accent syndrome (FAS).

The evidence that accent is extremely relevant both socially and linguistically is reflected by an increased interest of neuroscientists. An important aspect to advance our understanding on the neuroscience of accent is to gain further knowledge on the large-scale set of neural structures that modulate the reception and production of accents. Several populations are germane for the study of accent. One special group is composed of healthy late learners of a second language; another group consists of subjects who are unable to acquire their native pronunciation and instead speak with a foreign accent. Finally, other cases of interest for this Research Topic are patients with accent changes due to psychiatric disorders (psychogenic FAS) or neurological conditions and its variants (neurogenic FAS). Although pathological changes in accent have been considered rare, a growing number of cases are appearing in the literature. In the past decade, neuroscience has made a huge leap in developing new instrumental techniques for studying speech production (e.g., computer-assisted pronunciation training systems) and many labs worldwide are also using modern neuroimaging to explore the neural mechanisms underlying accent.

## Contributions to the current research topic

The 13 articles compiled in this Frontiers Research Topic bring together experimental and theoretical research that links the brain with the phenomena of accent processing and accent change in second language learning as well as in neurological and psychiatric patients. We believe that the full list of papers provides a comprehensive update of the current state-of-the-art on accent in normal and pathological conditions.

## Studies in healthy subjects

### New models of accent processing

The neural basis of accent is a hot topic in neuroscience research, yet so far there are only a handful of theories of how the brain learns a new accent. Simmonds takes advantage of the theory proposed by Jarvis (2004) on vocal motor learning in songbirds and humans. In this hypothesis and theory article, Simmonds proposes that accent learning depends on the early development of variability in the networks governing the production of each speech sound, an argument which favors accurate acquisition of native-like pronunciation of different languages. She concludes that the vocal learning pathway is less susceptible to variability in late learners of a second language (a sensitive period) than in early learners, because in the latter group this pathway is easily recruited allowing rapid accent learning. In their contribution, Adank et al. examine the neural bases of accented-speech perception. This mini-review aims to integrate the neural architecture of processing accented speech in a single model that incorporates key neural areas dealing with auditory and phonological processing, sensorimotor mapping, and cognitive control processes. Together, Simmonds and Adank et al.'s provocative proposals shed a new light on the production and perception of accent. Their viewpoints may open new avenues in the study of accent acquisition in healthy late L2 learners as well as in the remediation of pathological changes in accent.

### Learning a new accent

Christiner and Reiterer investigate the influence of mastering musical skills (as instrumentalists or as vocalists) on the ability to imitate a foreign accent. They show that both instrumentalists and vocalists outperform non-musicians and, not surprisingly, vocalists show superior performance than instrumentalists. This study suggests that intensive vocal and singing training may accelerate foreign accent acquisition processes. Two articles in the current Research Topic used functional magnetic resonance imaging (fMRI) or event-related potentials (ERPs) to assess the neural correlates of accent production and perception. Ghazi-Saidi et al. tested naming of phonologically and semantically similar words (cognates) across two languages (French: “piano”; Spanish: “piano”) using experimental linguistic and neuroimaging methods. They show that the native speakers of French struggled to produce cognates with the Spanish accent although L2 lexical learning was consolidated at the phonological and semantic levels. Note that attempts to produce cognates with new accents were cognitively and anatomically demanding as *f* MRI revealed upregulation of the left dorsal insula, a cortical region which plays key role in accent processing. Romero-Rivas et al. evaluate changes in real time processing of native- and foreign-accented speech with ERPs during language comprehension in healthy subjects. This study reveals fast compensatory processing modifications (lexical-semantic levels, linguistic reanalysis) in signal amplitude after brief exposure to foreign-accented speech.

### Feedback and accent learning

Banks et al. compare the role of audio-only and audio-visual speech recognition on perceptual adaptation in a large sample of healthy subjects. Contrary to predictions and although recognition of the novel accent using audio-visual speech cues was better than recognition on the basis of audio-only cues no differences were found in perceptual gains between the two modalities. Therefore, more is not always better, at least in novel accent perceptual adaptation. Katz and Mehta used real-time visual feedback of tongue movements with an interactive 3D visualization system based on electromagnetic articulography. They show that this method strengths learning of non-native speech sounds in healthy speakers. Hopefully, “tongue reading” using computer-assisted pronunciation training opens new avenues for L2 accent learning as well as for the improvement of several speech production disorders (stuttering, apraxia of speech, FAS). The conclusions of these two studies together with data from Christiner and Reiterer seem to support the view that perception alone, be it auditory or audio-visual, does not suffice for successful accent learning. On the contrary, it seems that intensive vocal practice (e.g., as in vocalists) and visual feedback of motor movements production (e.g., through a tongue avatar) may facilitate accent learning and imitation.

## Studies in patients with changes in accent

The increased number of reports on FAS during the past decade (Gurd and Coleman, 2006; Moreno-Torres et al., 2013) have led to a better definition of the three different types of FAS (developmental, psychogenic, and neurogenic; Verhoeven and Mariën, 2010). Several cases describing these subtypes and included in the Research Topic are summarized below.

### Developmental foreign accent syndrome

Keulen, Mariën, Wackenier, et al. report the case of an adolescent male with developmental FAS (DFAS) who did not show any familial antecedents of developmental disorders nor an abnormal personal psychiatric evaluation or cognitive testing except for impaired executive functions (non-verbal planning). A functional neuroimaging study with single photon emission computerized tomography (SPECT) showed a significant decrease of bloodflow on the medial prefrontal and lateral temporal regions bilaterally. The authors examine the boundaries between DFAS and DAS (developmental apraxia of speech) and since hypoperfusion approached statistical significance in cerebellum as well, they suggest that both disorders might be related to dysfunction in cerebro-cerebellar connections. Berthier et al. describe two adult males who presented with long-standing mild DFAS and internalizing psychiatric disorders (obsessions, anxiety, social phobia) which may suggest a psychogenic origin. Nevertheless, both subjects showed structural brain anomalies (venous malformation and expanded perivascular spaces) and diffusion tensor imaging additionally disclosed microstructural abnormalities in speech and emotion regulation networks. These results emphasize the need to use modern neuroimaging methods to detect subtle brain abnormalities in cases with a provisional diagnosis of psychogenic FAS (PFAS).

### Psychogenic foreign accent syndrome

Keulen et al. report three studies on PFAS (Keulen, Verhoeven, Bastiaanse, et al., Keulen, Verhoeven, De Page, et al., Keulen, Verhoeven, De Witte, et al.). In a review article, Keulen, Verhoeven, De Witte, et al. examine the extant literature (1907–2014) on the psychogenic subtype. This paper provides clues for its diagnosis in clinical practice and defends the relevance of classifying psychogenic cases as belonging to an independent category. Whitaker (1982) coined the term “foreign accent syndrome” and set out the initial criteria for its diagnosis in neurological cases. Keulen, Verhoeven, Bastiaanse, et al. consider that this early diagnostic recommendations were too restrictive and claim that a set of broader inclusion criteria is desirable to incorporate psychogenic cases. Finally, Keulen, Verhoeven, De Page, et al. report a new case of PFAS in a patient with head trauma. The absence of gross structural brain damage on neuroimaging coupled with the presence of a complex neuropsychiatric disorder lead the authors to assign the label of psychogenic. Cases like this revive the debate centered on the psychogenic and organic origins of FAS.

### A new variant of neurogenic foreign accent syndrome

Variants of FAS have been described including changes in regional accent (e.g., from Parisian accent to Alsatian accent), stronger regional accent, and re-emergence of a previously learned and dormant regional accent. Berthier et al. describe a new variant of FAS in this Research Topic in three adult males who after recovering from Broca's aphasia lose their regional accent. This study shows that focal lesions in the middle part of the left motor cortex and adjoining regions seem to be crucial to alter the neural processes implicated in the production of regional accent features.

### Synthesis and directions for future research

The articles in this Research Topic reveal that accent is not merely accessory to language but it is rather a fundamental component of it. Acquiring a new accent at an early age is easy partly thanks to the great flexibility of the neural networks supporting accent learning. Nevertheless, the acquisition of a new accent after childhood is very difficult because of the well-known reduction in plastic capability of the networks underpinning vocal learning. This means that healthy subjects who want to learn a new language or those who suffer changes in their native accent (e.g., FAS) as a result of psychiatric and/or neurological disorders will require external support to acquire/recover native-like accent. Therefore, it is necessary to further increase our knowledge on the neuroscience of accent so as to develop new training strategies focused on accent.

## Author contributions

IMT and MB contributed to the design of the work and drafted the editorial. PM and GD revised the draft for important intellectual content and contributed with the interpretation of the work. IMT, PM, GD, and MB approved the final version to be published.

### Conflict of interest statement

The authors declare that the research was conducted in the absence of any commercial or financial relationships that could be construed as a potential conflict of interest.
